# P^3^DB 3.0: From plant phosphorylation sites to protein networks

**DOI:** 10.1093/nar/gkt1135

**Published:** 2013-11-15

**Authors:** Qiuming Yao, Huangyi Ge, Shangquan Wu, Ning Zhang, Wei Chen, Chunhui Xu, Jianjiong Gao, Jay J. Thelen, Dong Xu

**Affiliations:** ^1^Department of Computer Science, University of Missouri, Columbia, MO 65211, USA, ^2^Bond Life Sciences Center, University of Missouri, Columbia, MO 65211, USA, ^3^School of Communication and Information Engineering, Shanghai University, Shanghai 200444, People’s Republic of China, ^4^Department of Biology, Brandeis University, MA 02453, USA, ^5^Computational Biology Center, Memorial Sloan-Kettering Cancer Center, New York, NY 10065, USA and ^6^Department of Biochemistry, University of Missouri, Columbia, MO 65211, USA

## Abstract

In the past few years, the Plant Protein Phosphorylation Database (P^3^DB, http://p3db.org) has become one of the most significant *in vivo* data resources for studying plant phosphoproteomics. We have substantially updated P^3^DB with respect to format, new datasets and analytic tools. In the P^3^DB 3.0, there are altogether 47 923 phosphosites in 16 477 phosphoproteins curated across nine plant organisms from 32 studies, which have met our multiple quality standards for acquisition of *in vivo* phosphorylation site data. Centralized by these phosphorylation data, multiple related data and annotations are provided, including protein–protein interaction (PPI), gene ontology, protein tertiary structures, orthologous sequences, kinase/phosphatase classification and Kinase Client Assay (KiC Assay) data—all of which provides context for the phosphorylation event. In addition, P^3^DB 3.0 incorporates multiple network viewers for the above features, such as PPI network, kinase-substrate network, phosphatase-substrate network, and domain co-occurrence network to help study phosphorylation from a systems point of view. Furthermore, the new P^3^DB reflects a community-based design through which users can share datasets and automate data depository processes for publication purposes. Each of these new features supports the goal of making P^3^DB a comprehensive, systematic and interactive platform for phosphoproteomics research.

## INTRODUCTION

Phosphorylation is one of the most pervasive protein modification types in plants. Phosphorylation and dephosphorylation act as an important switch in signal transduction, chemical metabolism and other inter- or intra-cellular processes (1). In eukaryotes, *O*-phosphorylation (serine, threonine and tyrosine) predominates the landscape of protein phosphorylation. The burgeoning amount of experimental phosphorylation site data has necessitated the development of databases to warehouse these data and provide an essential infrastructure for the research community.

P^3^DB debuted in 2009 (2) when there was a need for depositing, requesting and sharing the wealth of experimental plant phosphorylation data beyond the reference plant Arabidopsis. Since then, P^3^DB has been actively developed and regularly updated with new datasets and features. Since the initial release of P^3^DB, high-quality phosphorylation sites in this database have accumulated at a rapid pace due to improvements in enrichment techniques and mass spectrometry [Supplementary Figure S1a and b]. Most of the datasets in the database came from large-scale experiments (MS/MS) (3), although several smaller datasets were also deposited. To help users analyze the proteome-wide phosphorylation data more systematically, the new P^3^DB 3.0 provides more information and annotations about phosphoproteins such as gene ontology, homolog, 3D structures, kinase and phosphatase families, protein–protein interactions (PPIs) and protein domains, together with protein–protein networks, kinase-substrate or phosphatase-substrate networks and domain co-occurrence networks (4).

Although plant phosphoproteomics has its origins in Arabidopsis, at present there are more experimentally-mapped phosphorylation sites in nonmodel plants. As discovery plant phosphoproteomics extends beyond model organisms it is desirable to integrate and compare the diversity of phosphorylation events to fully interrogate the possibilities of regulation in *Viridiplantae*. In this regard P^3^DB aims to be a resource for the entire plant community. P^3^DB not only collects user-suggested datasets, but also allows research groups to directly deposit their data for the whole community or to share within a group. At the same time, they can collaborate and interact through this platform.

Besides P^3^DB, there are a number of other useful web resources for phosphorylation or other PTM data. HPRD, the Human Protein Reference Database (5,6), covers a wide range of PTM data including phosphorylation. However, it is restricted to human. PhosphoSitePlus (7) provides resources for integrating signaling pathways, but it only includes data for human, rat and mouse. PhosphoELM (8) contains many kinase-specific data, but it is not developed for plant phosphorylation data either. PhosphAT (9) is a rich resource for phosphorylation exclusively for Arabidopsis. However, it does not cover other plant species. MASCP Gator (10) is also a comprehensive resource for proteomic data integration in Arabidopsis, but it does not host the data itself. Furthermore, none of the above web services provide any features for network-based analysis or community-based services. Thus, P^3^DB is a unique, complementary database to the current databases for its broad coverage of plant species, network-based data presentation and visualization, and community-based data services.

## MATERIALS AND METHODS

### Datasets

The datasets in P^3^DB 3.0 are curated from literature, online resources and in-house collaborations. With 32 studies (Supplementary Table S1) from nine plant species included, P^3^DB 3.0 now has 47 923 nonredundant phosphorylation sites in 16 477 phosphoproteins. *Arabidopsis thaliana* (contributing 30.15% to the phosphoproteins total data), *Medicago truncatula* (25.36%) and *Oryza sativa* (29.31%) are the three plants having the most phosphorylation data in P^3^DB (Supplementary Figure S1c).

P^3^DB also supports private datasets, which can be shared within research groups or with manuscript reviewers by a simple link that has password protection. These datasets can be merged to the public dataset after official acceptance of the publication or by user’s authorized release.

### Data quality criteria

Currently only *in vivo* experimental data are collected and archived in P^3^DB, except for the *in vitro* data obtained to identify kinase–client relationships, i.e. KiC assay (11,12) data. Most of the data are from high-throughput experiments from different laboratories using different instrumentation and data mining strategies; therefore, the quality of the data varies among different studies. P^3^DB employs a strict data selection criterion: False Discovery Rate (FDR) <1% and <15 ppm precursor mass accuracy, to make sure phosphopeptide identification is of high quality.

### Web services and interface

P^3^DB provides a user-friendly, interactive interface for data access. It is implemented by a back-end MySQL database, server-side PHP code and front-end Javascript and jQuery tools. The web services and interface follow the new definitions of HTML5 and CSS3, which ensure the smooth running on any html5-compliant systems including mobile devices. Cytoscape (13) JavaScript APIs are used in the new P^3^DB for displaying the network features.

### Ontology

The gene ontology hierarchical architecture is retrieved from the ontology website (14,15) and integrated into P^3^DB. The annotations of plant proteins are from TAIR (16) and Phytozome (17). The ontology data can be used for searching and browsing phosphoproteins in different functional categories on the website.

### Plant kinase and phosphatase family

The tree structure of the kinase and phosphatase families is obtained from PlantsP (18). The kinases or phosphatases from other data sources are mapped to the tree structure by their corresponding annotations. In the case that the kinase cannot be assigned to any of the subclasses, it will be assigned as an unknown class or general kinase class.

### Kinase-Client assay

The KiC (Kinase-Client) assay is a technology to identify the phosphorylation sites associated with a kinase activity using a mass spectrometry-based *in vitro* screening process (11,12). A typical KiC assay requires purified kinase and a peptide library after which phosphorylation is quantified by mass spectrometry. After the peptide is identified as a target of a certain kinase, the whole-length protein can be used to further validate the kinase-substrate relationship (12). The KiC assay is a systematic way to screen the kinase–client relationship and help construct kinase-substrate networks.

### Protein 3D structure

Protein structures are obtained from the Protein Data Bank (PDB) (19) with IDs mapped from the Uniprot (20,21). Some of the structures for Arabidopsis are predicted protein structures at Interactome 3D (22). Jmol (23) is used to visualize the tertiary structures.

### PPI network

The PPI data are collected from four major databases: Biogrid (24), Intact (25), DIP (26) and Mint (27). The PPI data in plants other than Arabidopsis are still very limited. The interaction data are visualized in networks, by calculating direct interactions or indirect interactions with intermediate nodes.

### Protein domain network

Domains are structure- and function-independent units in proteins. The kinase domains, phosphatase domains, substrate domains and phosphorylation recognition domains are very important for understanding the functions of phosphorylation events. These domains may be fused into a single peptide to facilitate the phosphorylation activities, such as tyrosine kinase receptors. Such fusions can be represented by a domain co-occurrence network, where two domains form a link if they occur in the same protein. The domain library (Pfam A 27.0) is downloaded from the Pfam website (28), together with the proteomic annotations.

## NEW AND UPDATED FEATURES

### Protein chart

**Figure 1.**
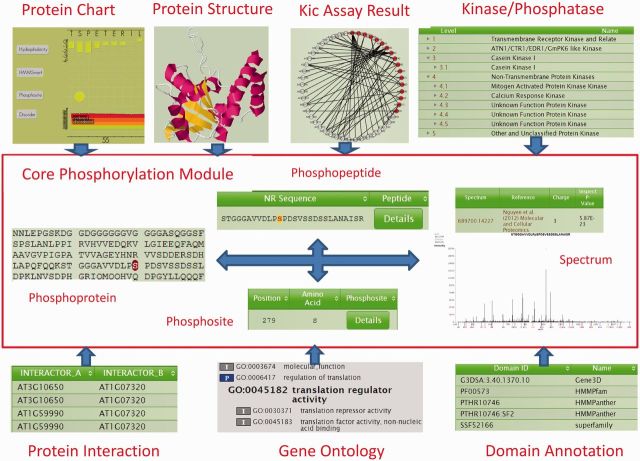
Module-based P^3^DB functionalities.

The protein chart provides information of local amino acid properties around phosphorylation sites, as shown in Figure 1. Phosphorylation sites are highlighted in green circles on the graph, and other related information is aligned to the phosphorylation sites, including predicted hydrophobicity values, involved domains and predicted disorder scores (29). Phosphorylation is overrepresented in disordered regions, as shown in previous studies (30–32). It is also useful to present the substrate domain so that functional information may be revealed, since the substrate domain can be a regulatory or recognition domain in the downstream signaling cascade. The hydrophobicity often has a low value at the phosphorylation site, which indicates that phosphorylation sites are generally more hydrophilic than the background. This is not surprising as phosphorylation sites are usually exposed to the surface and are in disordered regions. Thus, the protein chart may be helpful to build hypotheses based on protein function and amino acid properties. The flexible architecture allows P^3^DB to display more potential position-specific factors in the future like protein-binding sites or polarity.

### Orthologous sequence

Archiving the phosphoproteome of nonmodel plants in P^3^DB affords the plant biologist access to a larger, more complete resource of regulatory phosphorylation events. Questions about functional conservation can be directly queried through a new feature in version 3.0. Phosphorylation sites may or may not be conserved among sequences in the same orthologous group in plants (31). Orthologous groups identified by MCL (33) are used in P^3^DB. Orthologous sequences are aligned based on positions; thus, it is easy to see whether the phosphorylation site is conserved. However, if the expected phosphorylation event in the ortholog is not observed, it may be due to lack of the experimental evidence in our database.

### Gene ontology

**Figure 2.**
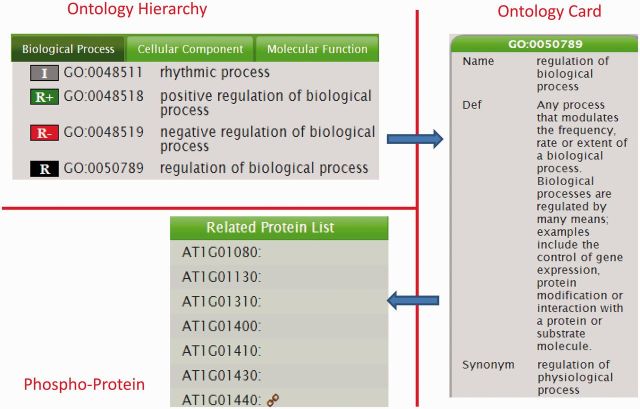
Ontology browser.

The ontology terms can be browsed or searched in a hierarchical view. The tree view and the ontology are cross-linked between the protein page and the ontology page. On the protein page, the gene ontology terms are listed to help the user understand the functions of the phosphorylated protein. On the ontology page, the tree structure shows the parental and sibling terms, which help the user to navigate among related terms, and phosphorylated proteins under each ontology term are listed explicitly (Figure 2).

### Kinase and phosphatase family

Kinases or phosphatases can be searched or browsed in a family tree view. This module is also cross-listed in the protein page. If a protein is annotated as a kinase, phosphatase or kinase substrate, the protein page will show this information. Notice that a protein can be both an enzyme and substrate. Every kinase or phosphatase is listed under the family tree view with a certain category. Some proteins are categorized to the unknown type or parent family due to lack of annotation.

### Site prediction portal

Although we only curate experimental datasets, the green plant specific prediction of phosphorylation sites is available in P^3^DB through an embedded link to Musite (31) in the protein page.

### Taxonomy browser

The taxonomy browser helps the user to explore the kingdom of *Viridiplantae* (green plants). The search page is also available for taxonomy information. The species page is cross-linked to phosphorylation datasets if available.

### PPI network

**Figure 3.**
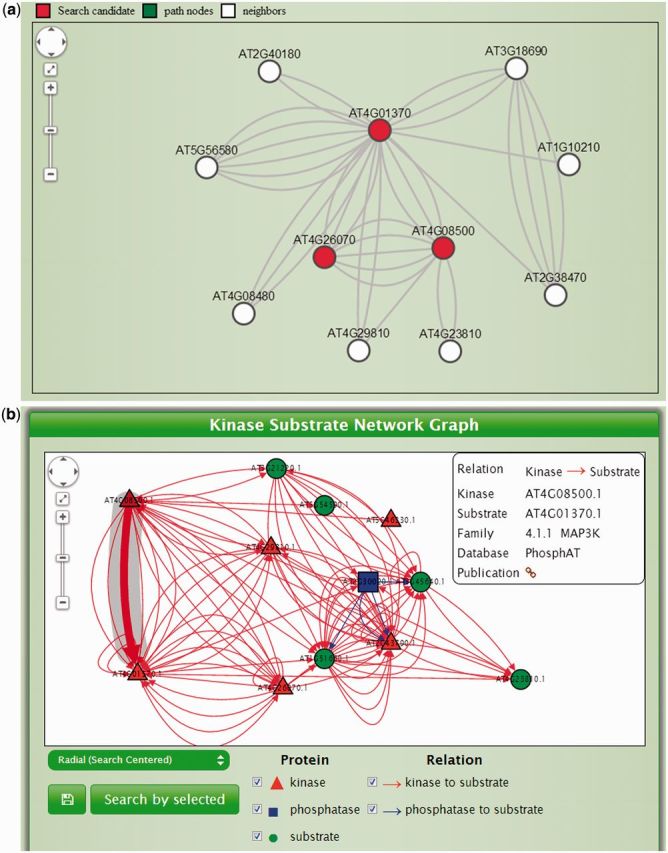
Cross-reference between PPI network and kinase/phosphatase networks. (**a**) PPI network; (**b**) kinase/phosphatase–substrate network.

Phosphoproteins in P^3^DB are also visualized in the context of PPI networks. Hypotheses in terms of the potential cause and effect can be constructed based on the PPI network and phosphorylation sites. For example, by searching interactions of AT4G26070, a MAPKK in Arabidopsis, the other two proteins AT4G01370 (MAPK4) and AT4G08500 (MAP3K) are found to have interactions with this MAPKK. The edge represents the data source validated or experimental verified relationships. In most cases, a single interaction is supported by multiple experiments or data resources. As seen from the interactions with these three MAP Kinases, there are 11 experimental evidences on them (34–37) (Figure 3a).

If two proteins do not interact with each other directly, P^3^DB will use the shortest path algorithm to find an interaction path that can connect these proteins. Based on the data and this algorithm, the pairwise linkage can be always found if it exists. This function is especially helpful to discover long-range relationships. The P^3^DB PPI network can also be expanded for each node with its neighboring interactions by simply clicking the node. The white nodes in Figure 3a represent proteins that are directly interacting with the three red proteins.

### Kinase and phosphatase-substrate network

An important data source for the kinase-substrate network is the emerging results from protein kinase and phosphatase client screens. For example, P^3^DB displays KiC assay results separately from other enzyme target data, since they may provide further details of phosphorylation sites. Meanwhile, the KiC assay results are also merged into the pool of the kinase-substrate network.

The kinase-substrate network is overlaid with the phosphatase-substrate network. Different colors of the nodes and edges are used to distinguish kinases and phosphatases, and phosphorylation and dephosphorylation. This network can provide complementary information to PPI networks. For example, in Figure 3b, the network obtained by searching from AT4G08500.1 and AT2G30020.1 contains kinases, a phosphatase and substrates with different colors and annotations. As a PPI partner seen in the previous example, AT4G01370.1 is also involved in this kinase-substrate network, which potentially reveals the MAP kinase cascade (38–41). Interestingly, AT2G30020.1 shows phosphatase activities in the network (42), which adds more information to what the PPI network can provide.

### Domain network

**Figure 4.**
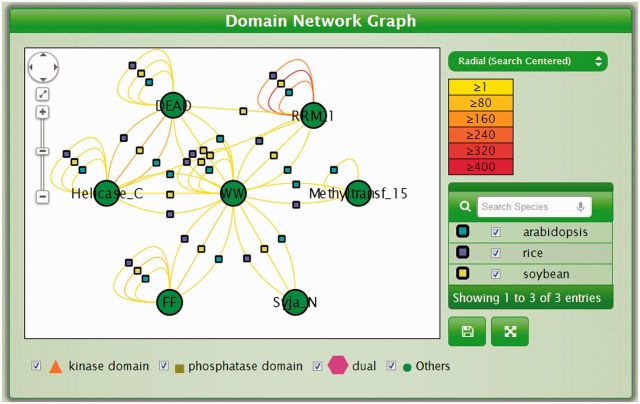
Domain co-occurrence network.

Domain co-occurrence networks can help reveal kinase-domain interactions, regulatory domains and the recognition domains in phosphorylation-signaling pathways. For example, WW is a recognition domain for those phosphorylated proteins containing the pSer/pThr-Pro motif through the local conformational change of proline isomerization (43). In the WW neighboring network, the RNA capping methyltransferase domains, the RNA-binding domains and the helicase domains can be found. This may indicate that the related phosphorylation events for WW recognized proteins may eventually change the protein expression by affecting the mRNA metabolism, splicing, ribosome assembly and translation initiation (44). In the domain co-occurrence network, the protein that contributes to the domain network is cross-listed. The thickness of the edge between domains indicates the number of proteins that contribute to the domain linkage (Figure 4). The domain network represents domain structures from different species with different colors, so that the conservation of the protein domains and functions can be viewed easily.

### Community-based user experience

#### Automated data curating system

**Figure 5.**
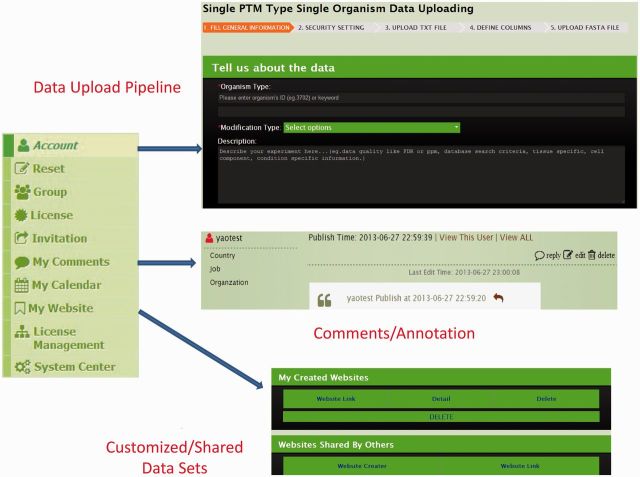
Community-based data curation.

Users can now upload data on their own, and P^3^DB will automatically generate a customized data repository site for publication purposes. Users can also delete their own datasets easily if they wish (Figure 5).

#### Data sharing and security control

Users can decide the access level of their own data by selecting public, private or shared within a group. The public user data can be merged to the main depository pool for general P^3^DB display if the data meet the quality requirement.

#### Annotation by comments

The protein data, phosphosite data and phosphopeptide data can be annotated and commented by users. Users can also reply or follow other users’ comments.

## CONCLUSION

P^3^DB is a comprehensive, systematic and interactive plant protein phosphorylation data resource. It helps researchers to analyze protein phosphorylation events across the plant kingdom, providing homology-based evidence for function. P^3^DB 3.0 provides several network-based data representation and visualization tools to view the functions and context of phosphorylation sites in multiple aspects, by integrating all the related information. The community-based design allows users to have better communication and control of their data in P^3^DB.

## SUPPLEMENTARY DATA

Supplementary Data are available at NAR Online.

## FUNDING

The National Science Foundation [DBI-0604439 and 1126992] and National Institutes of Health [R01-GM100701]. Funding for open access charge: National Institutes of Health [R01-GM100701].

*Conflict of interest statement.* None declared.
